# Investigation of Interferences of Wearable Sensors with Plant Growth

**DOI:** 10.3390/bios14090439

**Published:** 2024-09-11

**Authors:** Xiao Xiao, Xinyue Liu, Yanbo Liu, Chengjin Tu, Menglong Qu, Jingjing Kong, Yongnian Zhang, Cheng Zhang

**Affiliations:** College of Engineering, Nanjing Agricultural University, Nanjing 210095, China

**Keywords:** plant wearable sensor, plant growth interference, sensor design, plant health monitoring

## Abstract

Plant wearable sensors have shown exceptional promise in continuously monitoring plant health. However, the potential adverse effects of these sensors on plant growth remain unclear. This study systematically quantifies wearable sensors’ interference with plant growth using two ornamental species, *Peperomia tetraphylla* and *Epipremnum aureum*. We evaluated the impacts of four common disturbances—mechanical pressure, hindrance of gas exchange, hindrance of light acquisition, and mechanical constraint—on leaf growth. Our results indicated that the combination of light hindrance and mechanical constraint demonstrated the most significant interference. When the sensor weight was no greater than 0.6 g and the coverage was no greater than 5% of the leaf area, these four disturbances resulted in slight impacts on leaf growth. Additionally, we fabricated a minimally interfering wearable sensor capable of measuring the air temperature of the microclimate of the plant while maintaining plant growth. This research provides valuable insights into optimizing plant wearable sensors, balancing functionality with minimal plant interference.

## 1. Introduction

Wearable sensors, inspired by their great successes in human health monitoring [1,2,3,4], demonstrate huge potential in plant health monitoring [5,6,7,8]. Unlike traditional plant health monitoring methods [9,10,11], wearable sensors are intimately adhered to the epidermis of plants, offering minimally invasive, in situ, real-time, and long-term measurements [12,13,14]. The detection targets of plant wearable sensors not only include physiological/biochemical signs (such as leaf elongation [6,15,16], leaf temperature [17], hydration [18,19], biopotential [20,21,22], volatile organic compounds [17,23], and hormones [24,25]) that reflect plant health status, but they also include microclimatic factors (such as air temperature [6], humidity [26], and light [14]) that directly affect plant health.

**Figure 1:**
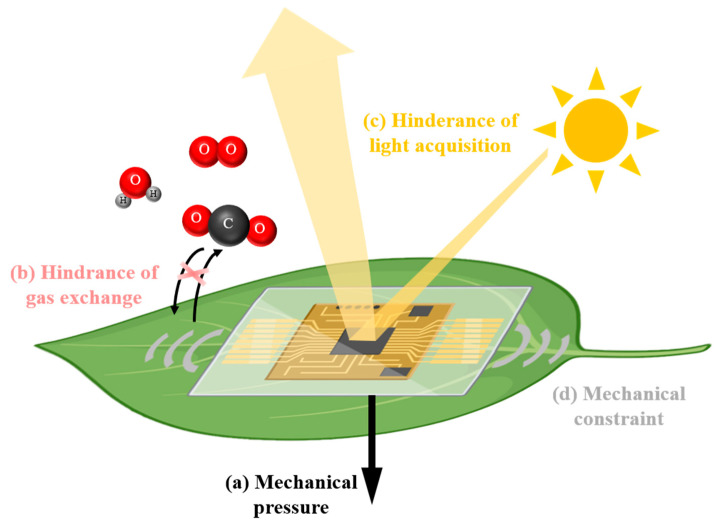
Interferences of wearable sensors with plant growth.

Despite the fact that wearable sensors are typically regarded as minimally invasive measurement tools, they may have adverse effects on plant health, which have been briefly considered in the reported works. As shown in Figure 1, the first disturbance is the mechanical pressure from the weight of the sensors. Mechanical pressure can compress cell walls, alter leaf morphology, and ultimately slow plant growth [27]. Therefore, researchers have been trying to fabricate lightweight wearable sensors, such as a leaf wearable sensor with a mass of only 17 mg and a pressure of only 170 μN on the leaf [14]. The second disturbance is the hindrance of gas exchange due to the limited breathability of the sensors. The intimate contact of wearable sensors could significantly hinder the gas flow of water vapor, oxygen, and carbon dioxide, which play essential roles in plant physiological activities. One plant wearable device has been endowed with a high breathability of 11.98 kg m^−2^ d^−1^ (for water vapor) using a porous substrate composed of nanofibers [28]. The third disturbance is the hindrance of light acquisition due to the limited transparency of the sensors. The coverage of wearable sensors on plant organs, especially on leaves, can block sunlight, which is the energy source of plants. Recently, a transparent plant e-skin was reported to have a transmittance over 85% (in the visible spectrum) using all-organic materials [29]. The last disturbance is the mechanical constraint due to the rigidity of the sensors. The size growth of plants is typically fast, especially compared to human beings, which could be constrained if wearable sensors cannot adapt to dimensional changes. Many researchers have been focused on improving the stretchability of plant wearable sensors [30,31,32,33]. For example, one hydrogel-based wearable device achieved a stretchability of 650% through a double-network design [30].

Although the aforementioned four disturbances of wearable sensors to plant growth have been widely acknowledged and many efforts have been made to optimize wearable sensors, the interference extents have not been quantified. This quantification is important mainly due to two reasons. First, it is complicated and expensive to lower the interference by optimizing wearable sensors [6,34]. The quantification of interference could help make a trade-off to improve cost efficiency. Second, the optimization strategies for these disturbances may be conflicting. For instance, a porous structure could increase breathability but decrease transparency [35]. The quantification of interference could help solve the main problem.

Herein, we systematically quantified the extent of interference of wearable sensors with plant growth. Polydimethylsiloxane (PDMS), a commonly used substrate for wearable sensors, was employed as a “simulator” with different properties to exert the four disturbances on the leaves of two common ornamental plants, *Peperomia tetraphylla* and *Epipremnum aureum*. The leaf length and width were recorded as growth indicators. It was found that the four disturbances all demonstrated interferences with leaf growth, and the interference extents increased as the amplitude of the four disturbances increased. However, when the weight was no greater than 0.6 g and the coverage (the area ratio of the simulator to leaf) was no greater than 5%, except the combination of the hindrance of gas exchange and mechanical constraint, a single hindrance of gas exchange or the combination of the hindrance of gas exchange and hindrance of light acquisition did not obviously interfere with leaf growth. Based on the analysis, a minimally interfering wearable sensor was fabricated. The wearable sensor was able to measure the air temperature of the microclimate of the plant and showed little interference with plant growth. This study can promote the development of plant wearable sensors.

## 2. Experimental Section

### 2.1. Cultivation of Peperomia Tetraphylla and Epipremnum Aureum

The plants were cultured by hydroponics in a controlled environment (20 ± 3 °C, 75 ± 5% RH). The main nutrient contents of the nutrient solution are N 686 mg/L, K_2_O 294 mg/L, P_2_O_5_ 294 mg/L, MgO 273 mg/L, and 4 mg/L (rounded to the nearest integer) other trace elements (such as Fe, Ca, B, Mn, and Zn). The plants were cultured in the nutrient solution and purified water alternately every 7 days to avoid overnutrition. An array of LEDs was used to ensure that each leaf received similar intensity of light (Figure S1).

### 2.2. Data Acquisition and Processing

The leaf length and width were measured daily using a vernier caliper. The leaf length was measured from the leaf base to the leaf tip, excluding the petiole. And the leaf width was defined as the longest extension of any two points on the leaf edge perpendicular to the midvein. The TRIMMEAN function was used to process the data. This method involves removing 2 maximum values and 2 minimum values from 10 groups of sample data and averaging the remaining 6 groups of samples.

### 2.3. Preparation of PDMS Simulators

PDMS precursors (NO. 705) were purchased from Shenzhen Guoyuan Technology Co., LTD. Part A and part B precursors were mixed in a mass ratio of 1:1. The mixture was thoroughly stirred and poured into homemade molds, followed by a 5 h solidification. The solidified samples were cut into the designed size to serve as PDMS simulators.

### 2.4. Fabrication of Wearable Temperature Sensor

Polyimide (PI) films (50 µm thick) were irradiated into laser-induced graphene (LIG) using a CO_2_ laser engraving machine (10.6 µm wavelength, 200 µm beam size, 9.3 W power, and 30 mm s^−1^ speed). The PDMS precursor mixtures were poured on the LIG. After 48 h, the solidified PDMS/LIG was peeled off as a wearable temperature sensor.

### 2.5. Characterization of Sensing Performances

The wearable temperature sensor was placed in an incubator and connected to a digital LCR meter (VICTOR 4092 A). The temperature and humidity in the incubator were tuned to the designed values while the resistance of the sensor was recorded by the LCR meter.

## 3. Results and Discussion

### 3.1. Experimental Design

The overall design principle aimed to simulate the real case as closely as possible in a convenient manner. To investigate the interference extents of wearable sensors to plant growth, the experiment was designed from three aspects, i.e., disturbances of wearable sensors, indicators of plant growth, and plant species.

For disturbances of wearable sensors (Figure 1), it was not necessary to use a complete wearable sensor to exert the disturbances. Alternatively, we only used the substrate of the wearable sensors as the simulator because it is the substrate that directly contacts the plant. The sensor can use a variety of materials as the sensitive layer/electrode layer (e.g., graphene, gold, and oxide semiconductor), but most of the substrate is PDMS, a silicone elastomer that is commonly used as the substrate for wearable sensors owing to its mechanical flexibility, chemical stability, and biocompatibility [6,18,36,37]. The interference extents of the four disturbances were studied by altering the properties of the PDMS, as explained in the following sections.

For indicators of plant growth, we selected leaf length and width. The reason is that the change in leaf area directly reflects the growth status of plants, and leaf area can be calculated based on a combination of leaf length and width [38,39]. Since the leaf length has a larger change amplitude than the leaf width but their change trends are consistent, we mainly discuss the extent of interference of wearable sensors on the leaf length in the main manuscript, while the effects on the leaf width are summarized in the Supporting Information.

For plant species, we selected *Peperomia tetraphylla* and *Epipremnum aureum*, two common indoor foliage plants. They are highly survivable and compatible with hydroponics, which can not only accurately control the plant environment but also reduce soil-borne diseases and pests. In addition, their leaves are relatively large, flat, and regular (orbicular or ovate), resulting in high convenience for size measurement.

### 3.2. Interference of Mechanical Pressure

First, PDMS simulators with cylindrical structures were prepared. To simulate different mechanical pressures induced by different weights, the heights of the cylinders were tuned while the base areas were fixed (radius = 5 mm). The reason for this is that the different base areas could result in crosstalk due to the hindrance of gas exchange or light acquisition. The PDMS simulators with the weights of 0.3 g, 0.6 g, 0.9 g, 1.2 g, and 1.5 g were gently placed on different plant leaves. When the weight was larger than 1.5 g, the PDMS simulator fell from the leaves because its gravity is larger than the self-adhesive force of PDMS. It is worth noting that the self-adhesive force is very weak, which could not constrain the size growth of leaves.

**Figure 2:**
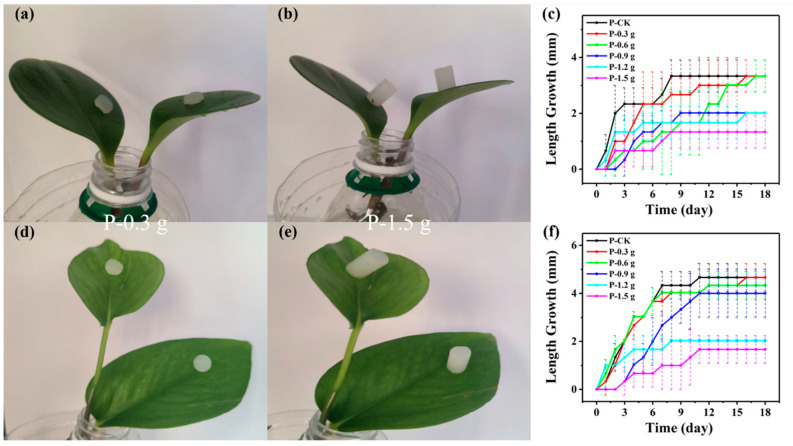
Interference of mechanical pressure on plant leaf length growth. *Peperomia tetraphylla* leaves with PDMS simulators attached, carrying weights of (**a**) 0.3 g and (**b**) 1.5 g. (**c**) The length growth of *Peperomia tetraphylla* leaves bearing different weights. *Epipremnum aureum* leaves with PDMS simulators attached, carrying weights of (**d**) 0.3 g and (**e**) 1.5 g. (**f**) The length growth of *Epipremnum aureum* leaves bearing different weights (the dashed lines represent error bars).

The optical photos of the *Peperomia tetraphylla* experimental groups are shown in Figure 2a,b and Figure S2a–c. The leaf length growth (compared to Day 0) of the experimental and control (P-CK) groups was recorded every day and is plotted in Figure 2c. Compared to the P-CK group, the length growth of the P-0.3 g and P-0.6 g groups showed similar rising trends. The P-0.3 g and P-0.6 g groups reached the same maximum length growth (3.3 mm) at Day 16 and Day 17, respectively, which was reached by the P-CK group at Day 8. The P-0.9 g and P-1.2 g groups displayed a smaller maximum (2.0 mm). For the P-1.5 g group, the maximum length growth was only 1.3 mm, less than the half of the P-CK group. Similar variation trends existed in the leaf width growth (Figure S2c).

For *Epipremnum aureum* (Figure 2d,e and Figure S2e–g), the P-0.3 g group reached the same maximum as the P-CK group (4.7 mm), while the P-0.6 g group reached a slightly smaller maximum (4.3 mm). For the rest of the groups, the heavier the simulator was, the smaller the maximum was. The changes in the leaf width growth were consistent with the length growth (Figure S2h).

In general, the leaf growth maximums of *Peperomia tetraphylla* and *Epipremnum aureum* gradually decreased with the weights of the simulators in the range of 0.3–1.5 g. When the weight was no larger than 0.6 g, the interference extent was slight.

### 3.3. Interference of Hindrance of Gas Exchange

To simulate the hindrance of gas exchange, there are two strategies. The first one is to use PDMS simulators with the same area but different breathability and to investigate the relationship between the leaf growth and the breathability. However, tuning the breathability is complicated not only for the PDMS simulators but for the real wearable sensors [40]. The other strategy is using PDMS simulators with different areas but the same breathability. Here, we directly used the pristine PDMS, which is often used as a coating to insulate the air [41]. To exclude the crosstalk from mechanical pressure, the weights of the PDMS simulators were unified at 0.6 g, which ensures that the areas of the PDMS simulators could be tuned to cover 5%, 10%, 20%, 60%, and 100% of the leaf area.

**Figure 3:**
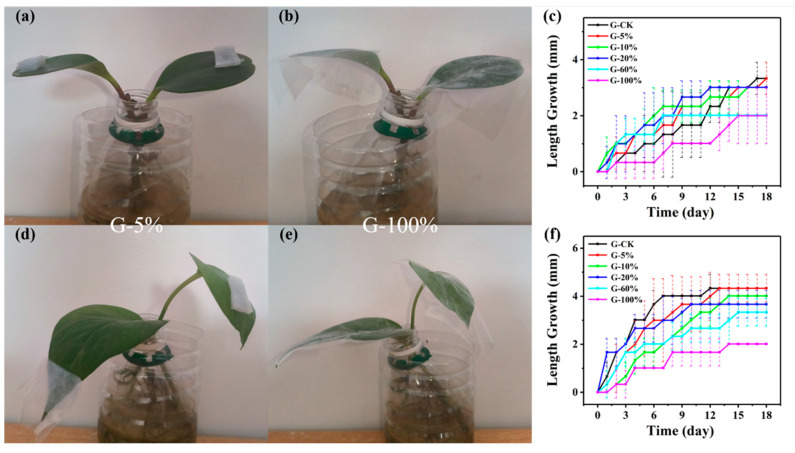
Interference of hindrance of gas exchange on plant leaf length growth. *Peperomia tetraphylla* leaves covered by non-breathable PDMS simulators with coverages of (**a**) 5% and (**b**) 100%. (**c**) The length growth of *Peperomia tetraphylla* leaves covered by non-breathable PDMS simulators with different coverages. *Epipremnum aureum* leaves covered by non-breathable PDMS simulators with coverages of (**d**) 5% and (**e**) 100%. (**f**) The length growth of *Epipremnum aureum* leaves covered by non-breathable PDMS simulators with different coverages (the dashed lines represent error bars).

The optical photos of the *Peperomia tetraphylla* experimental groups are shown in Figure 3a,b and Figure S3a–c. The G-CK and G-5% groups reached the same maximum length growth (3.3 mm) at Day 17 and Day 18, respectively (Figure 3c). The groups of G-10% and G-20% showed a slightly smaller maximum (3.0 mm) than G-CK. The maximum length growth of G-60% and G-100% was the same, 2.0 mm. Similar variation trends existed in the leaf width growth (Figure S3c).

For *Epipremnum aureum* (Figure 3d,e and Figure S3e–g), only the G-5% group demonstrated the same maximum length growth (4.3 mm) as the G-CK group, while the other groups demonstrated a decreasing maximum length growth from 4.0 mm to 2.0 mm as the coverage increased from 10% to 100%. Certainly, the growth rates also decreased as the coverage increased. For the leaf width growth, almost all groups kept the similar change trends with the length growth, except that the G-100% group did not display obvious increments in the experimental period (Figure S3h).

In summary, the leaf growth maximums of *Peperomia tetraphylla* and *Epipremnum aureum* gradually decreased with the coverage in the range of 5–100% when the leaves were covered by non-breathable PDMS simulators. When the coverage rate was no larger than 5%, the disturbance to *Peperomia tetraphylla* and *Epipremnum aureum* can be ignored.

### 3.4. Interference of Hinderance of Light Acquisition

Similar to the experimental design for the hindrance of gas exchange, the hindrance of light acquisition can be studied using PDMS simulators with either varying transparency or with varying cover area. We still chose the latter approach. The pristine PDMS is highly transparent, but the sensing layer and the electrode layer of wearable sensors are not necessarily transparent [6,42]. To simulate the hindrance of light acquisition, carbon black was introduced to prepare opaque PDMS simulators. The opaque PDMS simulators (the weights were unified at 0.6 g as well) with coverages of 5%, 10%, 20%, 60%, and 100% were gently placed on different plant leaves. Considering that the coverage could not only hinder the light acquisition but also the gas exchange, the results of this section (L groups) were compared with the results of Section 2.3 (G groups).

**Figure 4:**
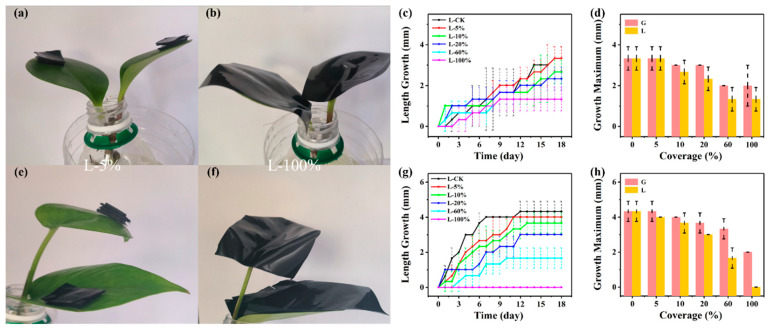
Interference of hindrance of light acquisition on plant leaf length growth. *Peperomia tetraphylla* leaves covered by non-breathable and opaque PDMS simulators with coverages of (**a**) 5% and (**b**) 100%. (**c**) The length growth of *Peperomia tetraphylla* leaves covered by non-breathable and opaque PDMS simulators with different coverages. (**d**) Comparison of the maximum length growth of *Peperomia tetraphylla* leaves covered by non-breathable and opaque PDMS simulators (L groups) and by non-breathable PDMS simulators (G groups). *Epipremnum aureum* leaves covered by non-breathable and opaque PDMS simulators with coverages of (**e**) 5% and (**f**) 100%. (**g**) The length growth of *Epipremnum aureum* leaves covered by non-breathable and opaque PDMS simulators with different coverages. (**h**) Comparison of the maximum length growth of *Epipremnum aureum* leaves covered by non-breathable and opaque PDMS simulators (L groups) and by non-breathable PDMS simulators (G groups) (the dashed lines represent error bars).

For *Peperomia tetraphylla* (Figure 4a,b and Figure S4a–c), as shown in Figure 4c, there was an almost noteless difference between L-CK and L-5% in terms of the maximum (3.3 mm). For the rest of the groups, they showed differences in the growth maximums, which decreased from 2.7 mm to 1.3 mm as the coverage increased from 10% to 100%. To distinguish the influence of the hindrance of light acquisition from that of gas exchange, the maximum leaf length growth of the G groups and L groups was compared. As shown in Figure 4d, the maximum growth of the G-5% and L-5% groups was the same, and very close to CK. As the coverage increased, the maximum growth decreased, and the L groups decreased more compared to G groups. For width growth, the leaves were not very susceptive to the hindrance of light acquisition (Figure S4d) or the hindrance of light acquisition and gas exchange (Figure S4e) unless the coverage was larger than 60%.

For *Epipremnum aureum* (Figure 4e,f and Figure S4f–h), the L-5%, L-10%, L-20%, and L-60% groups all demonstrated smaller growth maximums than L-CK, and the decreasing amplitudes rose with the coverage (Figure 4g). It is noted that L-100% did not show any growth during the experimental period. Compared to the G groups, the L groups had larger decreasing amplitudes as the coverage increased (Figure 4h). The changes in the leaf width growth were consistent with the length growth (Figure S4i,j).

In general, the leaf growth maximums of *Peperomia tetraphylla* and *Epipremnum aureum* gradually decreased with increasing coverage in the range of 5–100% when the leaves were covered by non-breathable and non-transparent PDMS simulators. When the coverage area was no larger than 5%, the interference was very slight.

### 3.5. Interference of Mechanical Constraint

In addition to the mechanical pressure induced by weights, wearable sensors can exert mechanical constraint on leaves due to their rigidity. Here, we still studied the mechanical constraint using PDMS simulators with varying coverage rather than varying rigidity. In the above three sections, the PDMS simulators were gently placed on leaves and remained on leaves by the self-adhesive force of PDMS, which did not transmit the mechanical constraint to leaves. In contrast, in this section, the PDMS simulators are fixed on leaves using PDMS precursors as glues. In this way, the covered leaf area is mechanically constrained to a large degree. Also, considering that the coverage could not only exert the mechanical constraint but also the gas exchange, the results of this section (C groups) are compared with the results of Section 2.3 (G groups).

**Figure 5:**
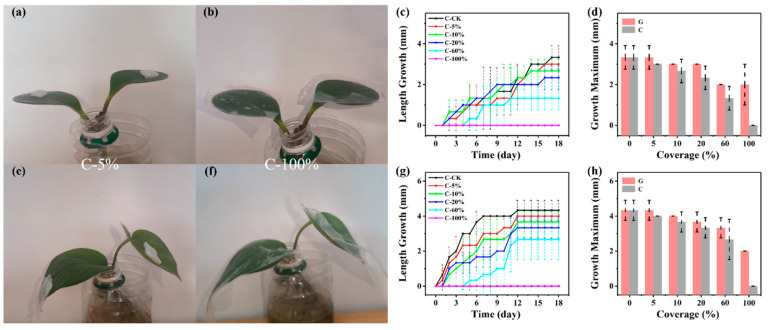
Interference of hindrance of mechanical constraint. *Peperomia tetraphylla* leaves mechanically constrained by non-breathable PDMS simulators with coverages of (**a**) 5% and (**b**) 100%. (**c**) The length growth of *Peperomia tetraphylla* leaves mechanically constrained by non-breathable PDMS simulators with different coverages. (**d**) Comparison of the maximum length growth of *Peperomia tetraphylla* leaves mechanically constrained by non-breathable PDMS simulators (C groups) and covered by non-breathable PDMS simulators (G groups). *Epipremnum aureum* leaves mechanically constrained by non-breathable PDMS simulators with coverages of (**e**) 5% and (**f**) 100%. (**g**) The length growth of *Epipremnum aureum* leaves mechanically constrained by non-breathable PDMS simulators with different coverages. (**h**) Comparison of the maximum length growth of *Epipremnum aureum* leaves mechanically constrained by non-breathable PDMS simulators (C groups) and covered by non-breathable PDMS simulators (G groups) (the dashed lines represent error bars).

*Peperomia tetraphylla* (Figure 5a,b and Figure S5a–c) and *Epipremnum aureum* (Figure 5e,f and Figure S5f–h) demonstrated very similar responses to mechanical constraint. For leaf length growth, the growth maximums of both plants decreased in all experimental groups, and the decreasing amplitudes increased with the coverage (Figure 5c,g). For the C-100% group, neither plant type showed any leaf length growth in the experimental period. Compared to the G groups, the C groups displayed smaller maximum growth at any coverage (Figure 5d,h).

Leaf width growth showed different change trends. First, the maximums did not reduce until the coverage exceeded certain thresholds, i.e., 10% for *Peperomia tetraphylla* (Figure S5d) and 5% for *Epipremnum aureum* (Figure S5i). Second, compared to the G groups, the leaf width growth of the C groups displayed limited reduction, especially for *Epipremnum aureum* (Figure S5e,j).

Thus, the length growth maximums of *Peperomia tetraphylla* and *Epipremnum aureum* gradually decreased with the coverage in the range of 5–100% when the leaves were gas-blocked and mechanically constrained by PDMS simulators.

Here is a summary of Section 3.2, Section 3.3, Section 3.4 and Section 3.5. Mechanical pressure, the hindrance of gas exchange, the hindrance of light acquisition, and mechanical constraint exerted by the PDMS simulators all demonstrated interferences to leaf growth of *Peperomia tetraphylla* and *Epipremnum aureum*. The interference extents increased as the amplitude of the four disturbances increased. But for *Peperomia tetraphylla*, it was sometimes found that its growth rate increases as the amplitude of the other three disturbances increases (Figure 3, Figure 4 and Figure 5). This may be due to the blocking of some stomata and thus a reduction the water loss of those stomata (i.e., transpiration), reducing the cooling effect of transpiration on the leaves [43]. This leads to a slight increase in leaf temperature, and the warmer environment may favor certain biochemical reactions that temporarily accelerate growth [44]. *Peperomia tetraphylla* may be more sensitive to temperature than *Epipremnum aureum*, so *Epipremnum aureum* does not have this phenomenon. However, when the weight was no larger than 0.6 g and the coverage was no larger than 5%, except for the combination of hindrance of gas exchange and mechanical constraint, the single hindrance of gas exchange or the combination of hindrance of gas exchange and hindrance of light acquisition did not obviously interfere with leaf growth.

### 3.6. Plant Wearable Sensor with Little Interference

**Figure 6:**
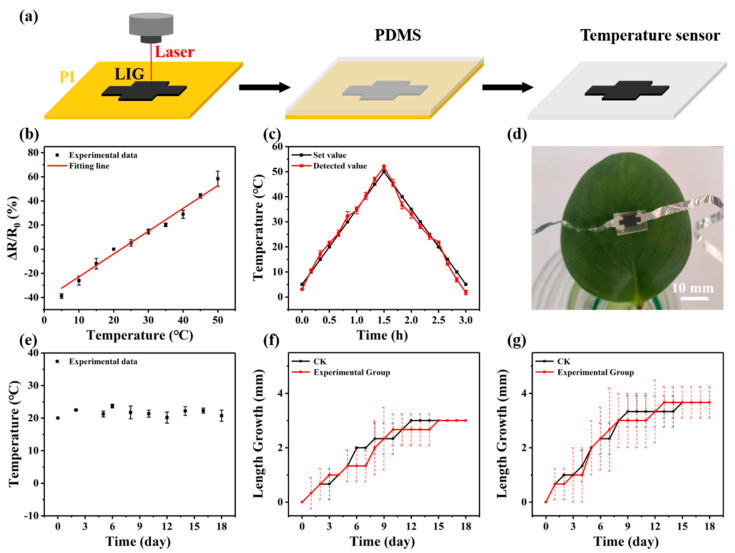
Plant wearable temperature sensor with little interference: (**a**) fabrication process, (**b**) sensing characteristic curve, (**c**) dynamic response curve, (**d**) digital image, and (**e**) continuous monitoring results of the wearable sensor. The length growth of (**f**) *Peperomia tetraphylla* leaves and (**g**) *Epipremnum aureum* leaves bearing the wearable sensor (the dashed lines represent error bars).

According to the above summary, we tried to fabricate a real wearable sensor with little interference to plant growth. LIG, a temperature-sensitive material [45,46], was selected to fabricate a wearable temperature sensor to monitor the microclimate of plants [15]. Specifically, LIG was first synthesized on a PI film via laser direct writing technology [45,47] and then transferred to a PDMS substrate (Figure 6a).

The sensing performances of the sensor were characterized in a plant incubator. As the temperature increased from 5 °C to 50 °C, the resistance variation ratio (ΔR/R_0_) of the sensor increased from −26.00% to 58.46%, where R_0_ was the resistance at room temperature (20 °C). This relationship could be fitted with the linear equation ΔR/R_0_ = 1.89T − 41.78 (R^2^ = 0.97), where T stands for temperature (°C). Thus, the sensor showed a sensitivity of 1.89%/°C and a linearity of 0.97. The mechanism of this change rule could be ascribed to the different thermal expansion coefficients of LIG and PDMS [48]. The dynamic measurement performance was also tested (Figure 6c). As the set temperature first increased from 5 °C to 50 °C and then decreased from 50 °C to 5 °C, the detected values of the sensor were highly consistent with the set values. Moreover, the wearable sensor displayed an excellent anti-humidity capability (Figure S6).

In addition to the good sensing performances, the wearable sensor had a small weight of 0.15 g and a small area of 1.5 cm^2^, which only accounted for 4.2% of a *Peperomia tetraphylla* leaf. After it was gently placed on the leaf (Figure 6d), it could continuously monitor the microclimatic temperature of the plant in the experimental period (Figure 6e). Moreover, the wearable sensor did not show significant interference on the leaf in terms of growth rate and growth maximum (Figure 6f and Figure S7a). This result was confirmed by the experiment performed on *Epipremnum aureum* leaves (Figure 6g and Figure S7b).

## 4. Conclusions

In summary, this study provides a comprehensive analysis of the extent to which wearable sensors interfere with plant growth, focusing on four primary disturbances. Through systematic experimentation with *Peperomia tetraphylla* and *Epipremnum aureum*, we found that the combination of light hindrance and mechanical constraint significantly impacted leaf growth. Our findings underscore the importance of optimizing sensor design to minimize these adverse effects. The development of a minimally interfering wearable sensor, as demonstrated in this research, represents a significant advancement in this field. This work not only enhances our understanding of plant–sensor interactions but also paves the way for more effective and less invasive plant monitoring technologies, ultimately contributing to sustainable agricultural practices.

## Data Availability

Data will be provided upon request from the corresponding author.

## Supplementary Materials

The following supporting information can be downloaded at https://www.mdpi.com/article/10.3390/bios14090439/s1, Figure S1: Plant growth environment; Figure S2: Interference of mechanical pressure on plant leaf width growth; Figure S3: Interference of hindrance of gas exchange on plant leaf width growth; Figure S4: Interference of hindrance of light acquisition on plant leaf width growth; Figure S5: Interference of hindrance of mechanical constraint; Figure S6: Resistance changes with relative humidity; Figure S7: Interference of the wearable temperature sensor on plant width growth.
