# Supplementary material for: Investigation of Interferences of Wearable Sensors with Plant Growth
Source: Biosensors (Basel). 2024 Sep 11;14(9):439. doi: 10.3390/bios14090439 (PMC11430609; doi:10.3390/bios14090439)
Supplement: Supplementary file 1 [file biosensors-14-00439-s001.zip › biosensors-3171182-supplementary.pdf]

Supplementary Information

Investigation of Interferences of Wearable Sensors with Plant Growth

Xiao Xiao †, Xinyue Liu †, Yanbo Liu, Chengjin Tu, Menglong Qu, Jingjing Kong, Yongnian Zhang * and Cheng Zhang *

College of Engineering, Nanjing Agricultural University, Nanjing 210095 and China.

* Correspondence: hczyn@njau.edu.cn (Y.Z.); zhangcheng@njau.edu.cn (C.Z.)

† These authors contribute equally to this work.

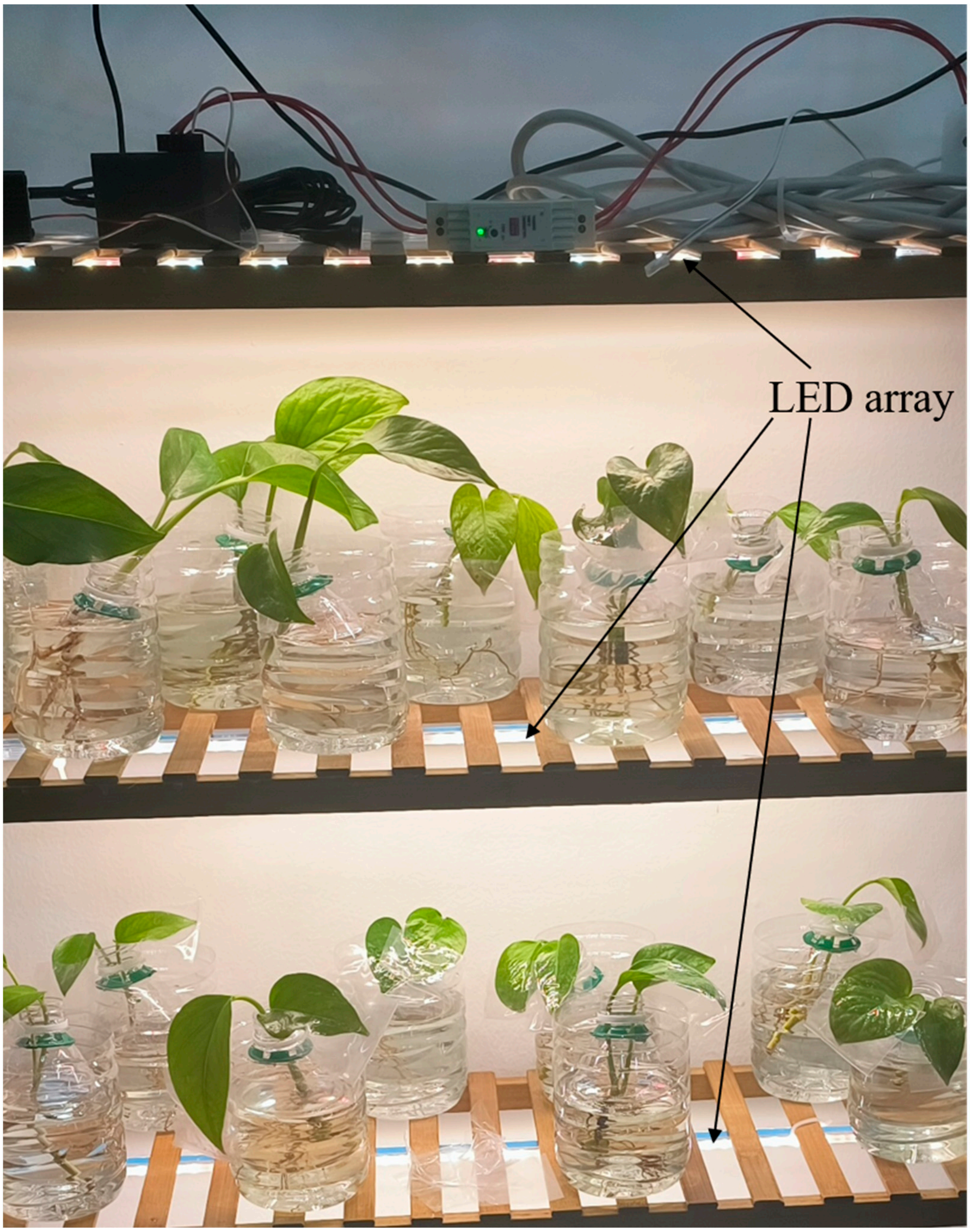

Figure S1. Plant growth environment.

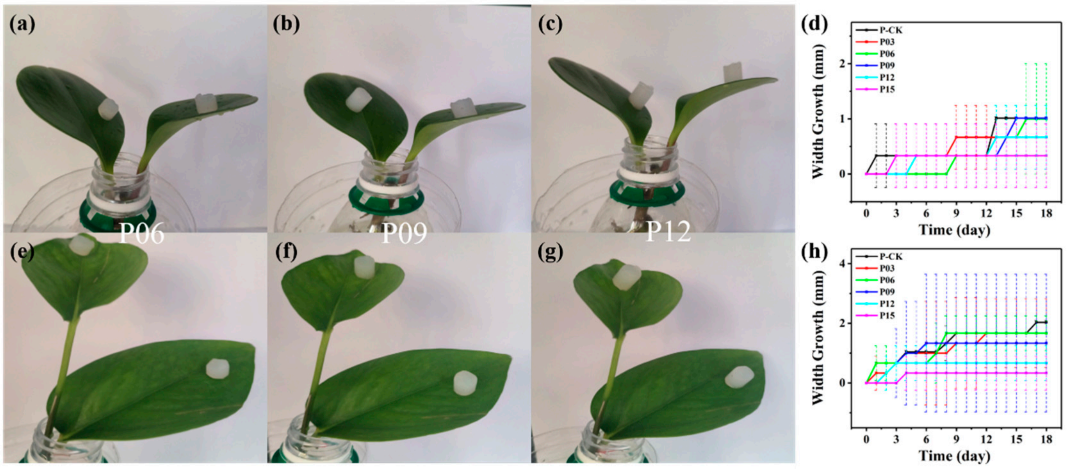

Figure S2. Interference of mechanical pressure on plant leaf width growth. *Peperomia tetraphylla* leaves with PDMS simulators attached, carrying weights of (a) 0.6 g, (b) 0.9 g, and (c) 1.2g. (d) The width growth of *Peperomia tetraphylla* leaves bearing different weights. *Epipremnum aureum* leaves with PDMS simulators attached, carrying weights of (e) 0.6 g, (f) 0.9 g, and (g) 1.2 g. (h) The width growth of *Epipremnum aureum* leaves bearing different weights.

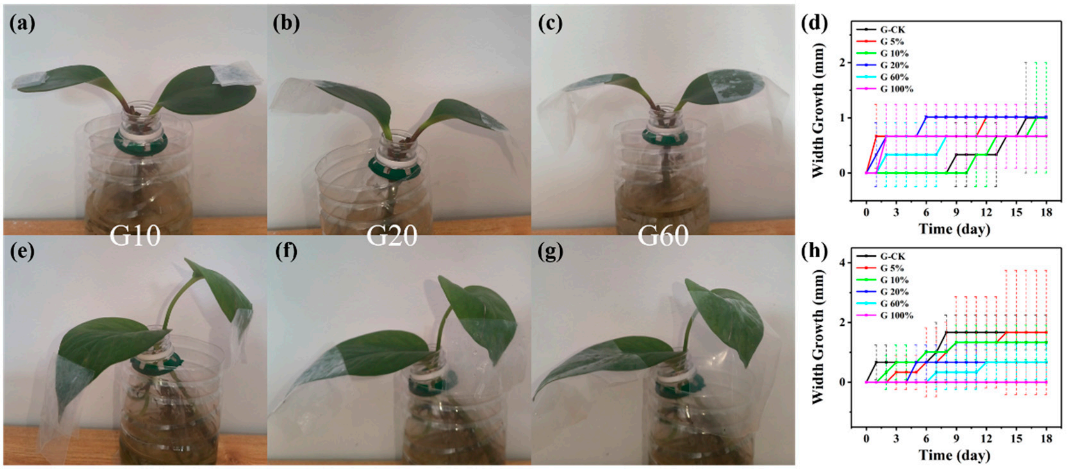

Figure S3. Interference of hindrance of gas exchange on plant leaf width growth. *Peperomia tetraphylla* leaves covered by non-breathable PDMS simulators with coverages of (a) 10%, (b) 20%, and (c) 60%. (d) The width growth of *Peperomia tetraphylla* leaves covered by non-breathable PDMS simulators with different coverages. *Epipremnum aureum* leaves covered by non-breathable PDMS simulators with coverages of (e) 10%, (f) 20%, and (g) 60%. (h) The width growth of *Epipremnum aureum* leaves covered by non-breathable PDMS simulators with different coverages.

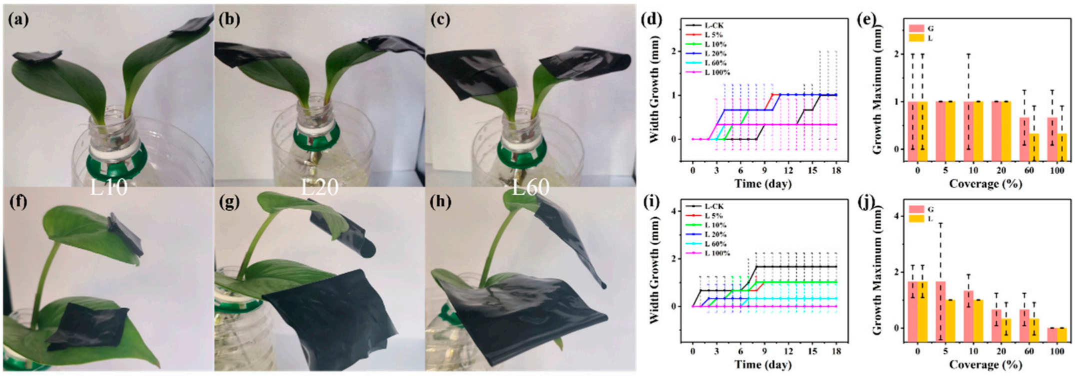

Figure S4. Interference of hindrance of light acquisition on plant leaf width growth. *Peperomia tetraphylla* leaves covered by non-breathable and opaque PDMS simulators with coverages of (a) 10%, (b) 20%, and (c) 60%. (d) The width growth of *Peperomia tetraphylla* leaves covered by non-breathable and opaque PDMS simulators with different coverages. (e) Comparison of the maximum width growth of *Peperomia tetraphylla* leaves covered by non-breathable and opaque PDMS simulators (L groups) and by non-breathable PDMS simulators (G groups). *Epipremnum aureum* leaves covered by non-breathable and opaque PDMS simulators with coverages of (f) 10%, (g) 20% and, (h) 60%. (i) The width growth of *Epipremnum aureum* leaves covered by non-breathable and opaque PDMS simulators with different coverages. (j) Comparison of the maximum width growth of *Epipremnum aureum* leaves covered by non-breathable and opaque PDMS simulators (L groups) and by non-breathable PDMS simulators (G groups).

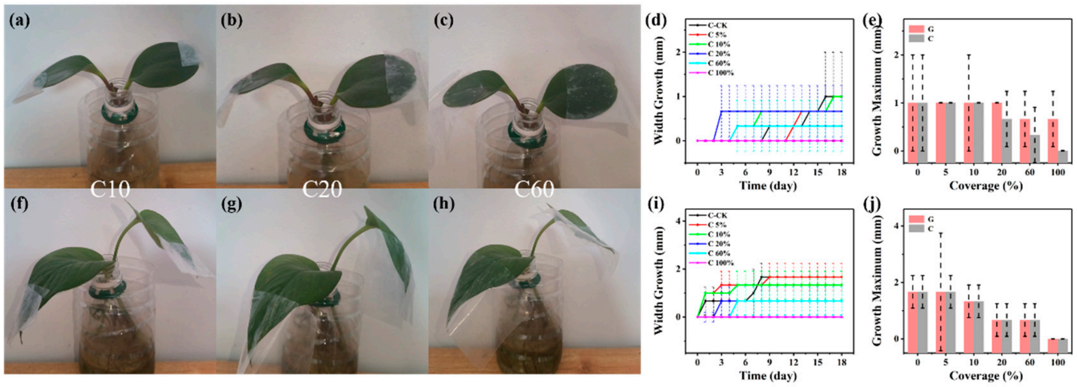

Figure S5. Interference of hindrance of mechanical constraint. *Peperomia tetraphylla* leaves mechanically constrained by non-breathable PDMS simulators with coverages of (a) 10%, (b) 20%, and (c) 60%. (d) The width growth of *Peperomia tetraphylla* leaves mechanically constrained by non-breathable PDMS simulators with different coverages. (e) Comparison of the maximum width growth of *Peperomia tetraphylla* leaves mechanically constrained by non-breathable PDMS simulators (C groups) and covered by non-breathable PDMS simulators (G groups). *Epipremnum aureum* leaves mechanically constrained by non-breathable PDMS simulators with coverages of (f) 10%, (g) 20%, and (h) 60%. (i) The width growth of *Epipremnum aureum* leaves mechanically constrained by non-breathable PDMS simulators with different coverages. (j) Comparison of the maximum width growth of *Epipremnum aureum* leaves mechanically constrained by non-breathable PDMS simulators (C groups) and covered by non-breathable PDMS simulators (G groups).

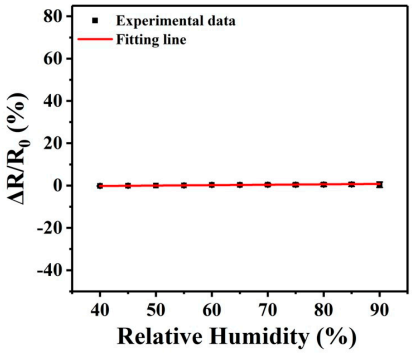

Figure S6. Resistance changes with relative humidity.

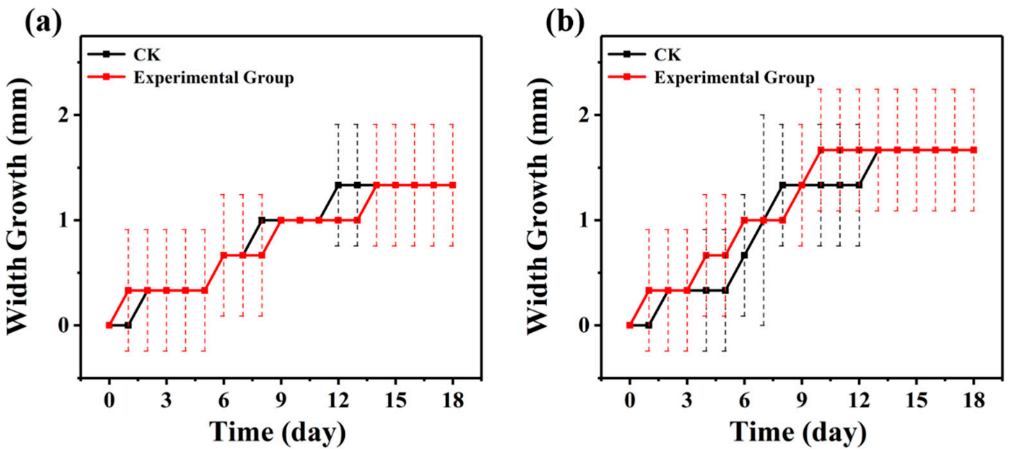

Figure S7. Interference of the wearable temperature sensor on plant width growth. The width growth of (a) *Peperomia tetraphylla* leaves and (b) *Epipremnum aureum* leaves bearing the wearable sensor.
